# Supplementary material for: Germination pretreatments to break hard-seed dormancy in Astragalus cicer L. (Fabaceae)
Source: PeerJ. 2016 Nov 3;4:e2621. doi: 10.7717/peerj.2621 (PMC5101587; doi:10.7717/peerj.2621)
Supplement: Supplemental Information 1 — Number of germinated seeds (out of 50) for each pretreatment, on a given day of the experiment. [file peerj-04-2621-s001.doc]

Table of raw data: Number of germinated seeds (out of 50) for each pretreatment, on a given day of the experiment.

| **Treatment day** | **0** | **6** | **10** | **13** | **17** | **20** | **24** | **27** | **33** |
| --- | --- | --- | --- | --- | --- | --- | --- | --- | --- |
| **Control** | 0 | 6 | 9 | 11 | 13 | 14 | 14 | 14 | 15 |
| **Hot water** | 0 | 0 | 0 | 0 | 0 | 0 | 0 | 0 | 0 |
| **Acid** | 0 | 6 | 13 | 17 | 17 | 17 | 17 | 17 | 17 |
| **Physical** | 0 | 9 | 31 | 35 | 37 | 37 | 37 | 37 | 37 |
| **Peroxide** | 0 | 6 | 7 | 10 | 10 | 12 | 13 | 13 | 13 |
| **Fire** | 0 | 0 | 0 | 0 | 0 | 0 | 0 | 0 | 0 |
